# Antimicrobial and Antibiofilm Activity of Cinnamon ( *Cinnamomum burmanii* ) Extract on Periodontal Pathogens—An *in vitro* study

**DOI:** 10.1055/s-0041-1742125

**Published:** 2022-04-18

**Authors:** Caesary Cloudya Panjaitan, Armelia Sari Widyarman, Rahmi Amtha, Tri Erri Astoeti

**Affiliations:** 1 Department of Public Health and Preventive Dentistry, Faculty of Dentistry, Trisakti University, Jakarta, Indonesia; 2 Department of Microbiology, Faculty of Dentistry, Trisakti University, Jakarta, Indonesia; 3 Oral Medicine Department, Faculty of Dentistry, Trisakti University, Jakarta, Indonesia; 4 Department of Public Health and Preventive Dentistry, Faculty of Dentistry, Trisakti University, Jakarta, Indonesia

**Keywords:** cinnamon, biofilm, toxicity, *Porphyromonas gingivalis*, *Aggregatibacter actinomycetemcomitans*

## Abstract

**Objective** The aim of this study was to analyze the antibiofilm effectiveness of cinnamon ( *Cinnamomum burmanii* ) ethanol extract against *Porphyromonas gingivalis* and *Aggregatibacter actinomycetemcomitans in vitro* .

**Material and** **Methods** Phytochemical tests were done to identify the cinnamon extract active substances. Furthermore, a crystal violet biofilm assay was used to analyze biofilm detachment after treatment with different concentrations (15, 10, 7.5, 5, and 2.5%) of cinnamon. Biofilm turbidity was measured at 595-nm wavelength using a microplate reader. Additionally, MTT assay was done to analyze the toxicity level of cinnamon extract on fibroblast cells.

**Statistical Analysis** The obtained data were analyzed for normality using the Kolmogorov–Smirnov test. The differences between each group were analyzed using a one-way analysis of variance statistical test, with a significance level of *p* <0.05.

**Results** The results showed that the ethanol extract of cinnamon contains active compounds of flavonoids, alkaloids, saponins, tannins, quinones, and terpenoids. MTT result showed the cinnamon extract to be nontoxic. The biofilm assay results showed that all cinnamon concentrations exhibited an antibiofilm effect against *P. gingivalis* and *A. actinomycetemcomitans* in all incubation time compared with a negative control ( *p* <0.05).

**Conclusions** Cinnamon extracts were effective in inhibiting biofilm of periodontal pathogens. Cinnamon extract might be useful as an alternative therapy for periodontal diseases. Future studies are still needed to confirm this result *in vivo* to analyze the efficacy of this extract as mouthwash.

## Introduction

Periodontal disease is a chronic inflammatory disease and most common oral condition of human population that affects approximately 20% to 50% around the world. 1 In Indonesia, based on basic health research data, the incidence of gingivitis and periodontitis in the general population was 23.4% 2 in 2013, and this rose to 74.1% in 2018. 3 Periodontitis is a destructive inflammatory disease of the supporting tissues of the teeth caused by specific microorganisms. 4 These microorganisms can damage the periodontal ligament and alveolar bone over time, with clinical manifestations of pocket formation, tooth mobility, loss of attachment, and gingival recession. 4 Four bacterial species are strongly associated with the initiation and development of periodontitis: *Porphyromonas gingivalis* , *Aggregatibacter actinomycetemcomitans* , *Tannerella forsythensis* , and *Prevotella intermedia* . 5 According to previous research, the dominant bacterial species in chronic periodontitis is *P. gingivalis* , with a prevalence of approximately 80.5%. 6 *A. actinomycetemcomitans* is dominant in aggressive periodontitis, with a frequency of approximately 90%, as well as in chronic periodontitis, where it is found in 21% of cases. 7 These bacteria replicate and form microcolonies, which irreversibly adhere to the epithelial surface and form a matrix consisting of extracellular polymeric substances called biofilms. These biofilms play a major role in the etiopathogenesis of periodontitis. 8

Periodontitis treatment includes mechanical and chemical biofilm control. Mechanical treatment involves tooth brushing, scaling, and root planing. 8 Chemical treatment involves mouthwashes and antibiotics, administered both locally and systemically. 9 According to a meta-analysis, the mouthwash chlorhexidine is effective in preventing biofilm formation. 10 However, research published in 2018 reported that chlorhexidine has a cytotoxic effect on normal cells in the oral cavity, in addition to clinical side effects, including tooth discoloration, oral mucosal irritation, and taste changes. 11 Long-term use of antibiotics term may also cause resistance to bacteria. Therefore, alternatives to chemical-based mouthwashes based on natural ingredients need to be developed.

One such natural ingredient that can be used as an alternative to chemical-based mouthwashes is cinnamon. More than 80,000 tons of cinnamon are produced each year in Indonesia. Thus, cinnamon is a readily available natural product in Indonesia that can be harvested easily and cost effectively. 12 Cinnamon spice is used as a flavor in food, as well as in scents. Cinnamon is reported to have health benefits, such as maintaining blood regulation. 13 The active compounds in cinnamon include flavonoids, alkaloids, saponins, tannins, quinones, and terpenoids, all of which are reported to have antibacterial, antibiofilm, and anti-inflammatory activities. 14

Gupta et al reported that gargling cinnamon extract with a concentration of 20% for 30 days reduced plaque and gingivitis clinically. 15 Other research reported that cinnamaldehyde, a compound in cinnamon, shows antibacterial activity and antibiofilm activity against *Staphylococcus aureus* (gram-positive bacterium) and *Escherichia coli* (gram-negative bacterium). 16 17 Previous studies focused on possible dental health benefits of cinnamon in the form of extracts, essential oils, and mouthwashes clinically and *in vitro* . 18 19 However, no studies have investigated the effect of ethanol extract of cinnamon on *P. gingivalis* and *A. actinomycetemcomitans* , which are the bacterial species mainly responsible for periodontitis. Thus, the aim of this study is to analyze the antibiofilm effectiveness of cinnamon ethanol extract against *Porphyromonas gingivalis* and *Aggregatibacter actinomycetemcomitans* as periodontal pathogen *in vitro* .

## Materials and Methods

### Cinnamon ( *Cinnamomum burmanii* ) Ethanol Extract Preparation

Cinnamon powder was collected from the Plant Research Institute of Spices and Medicines, Bogor, Indonesia (BALITTRO) and identified by the Indonesian Institute of Sciences. Approximately 100 g of the powder was macerated using 80% ethanol, with one part cinnamon powder to 10 parts of 80% ethanol. 20 The maceration procedure was performed for 3 × 24 hours, with occasional shaking of the solution. The solution was filtered using 0.1-mm Whatman filter paper. The solvent was evaporated using a rotary evaporator at 60°C until cinnamon ethanol extract was obtained. The extract was then placed in a closed container and stored at −20°C. The cinnamon ethanol extract was dissolved in 10% dimethyl sulfoxide and then diluted with distilled water into five concentrations (15, 10, 7.5, 5, and 2.5%).

### Phytochemical Tests

- Flavonoid test, as documented by Ejikeme et al in 2014: A total of 2 mL of cinnamon ethanol extract was added to 2 mL of diluted ammonia (NH _3_ ) and 1 mL concentrated sulfuric acid (H _2_ SO _4_ ) and shaken. A change in the color of the solution to yellow indicated the presence of flavonoid compounds 21 ;
- Alkaloid test, as reported by Usman et al in 2009: A total of 2 mL of Dragendorff's reagent (VWR Chemical, Radnor, Pennsylvania, United States) was added after 2 mL of ethanol extract was added with 1 mL of 1% hydrochloric acid (HCl), stirred in a water bath and filtered. A subsequent orange precipitate indicated the presence of alkaloid compounds. 22
- Saponin test, as proposed by Usman et al in 2009 with modifications: A total of 2 mL of cinnamon ethanol extract was added to 2 mL of distilled water and shaken vigorously vertically, followed by the addition of HCl. The formation of foam that remained stable in the solution after the addition of HCl indicated the presence of saponin compounds. 22
- Tannin test, according to Ejikeme et al in 2014 with modifications: A total of 2 mL of cinnamon ethanol extract was added to 2 mL of 1% ferric chloride (FeCl _3_ ) and then shaken. A change in the color of the solution to blackish brown indicated the presence of tannin compounds. 21
- Quinone test, in accordance to Krvavych et al in 2014: A total of 2 mL of cinnamon ethanol extract was added to 2 mL of concentrated sulfuric acid and then shaken. A change in the color of the solution to red indicated the presence of quinone compounds. 23
- Terpenoid test, as written by Usman et al in 2009: A total of 2 mL of cinnamon ethanol extract was added to 2 mL of acetic anhydride, shaken for homogenization, heated to boiling, cooled and followed by the addition of 1 mL of concentrated sulfuric acid along the tube walls. A visualized violet colored ring denoted the presence of terpenoid compounds. 22

Positive qualitative data (+) were obtained for each type of examination where there was a color change or reaction in accordance with the provisions after the sample or cinnamon ethanol extract was treated with the reagent. 24

### Cytotoxicity Test/3-(4,5-Dimethylthiazol-2-yl)-2,5-Diphenyltetrazolium Bromide (MTT) Assay on Fibroblast Cell Culture

The fibroblast cultured cells were grown using complete medium, i.e., Dulbecco's Modified Eagle Medium (DMEM) (Thermo-Fisher, Waltham, Massachusetts, United States) supplemented with 10% (v/v) fetal bovine serum with 100 IU/ mL penicillin and 100 ug/ mL streptomycin (Sigma-Aldrich, St. Louis, Missouri, United States) in a 175 cm ^2^ T-flask. The cells were maintained in 37°C with a 5 to 10% carbon dioxide (CO _2_ ) and humidified atmosphere during incubation.

To perform the MTT Assay, 100 μL of cell culture (2 × 10 ^5^ cells/ mL) was inserted into a 96-well plate, followed by incubation at 37°C for 48 hours. The remaining cell culture medium was discarded, and the cells were washed with 100 μL of sterile phosphate-buffered saline (PBS) (Oxoid, Hampshire, United Kingdom). The PBS was also discarded. The cells were then treated with cinnamon ethanol extract at concentrations of 15, 10, 7.5, 5, and 2.5%. In the study, 0.1% hydrogen peroxide (H _2_ O _2_ ) served as a positive control, and sterile distilled water served as a negative control. All treatments were repeated three times. The treated cells were then incubated at 37°C for 24 hours.

After incubation, the supernatant of each treated well was discarded, and the wells were washed with 100 μL of sterilized PBS. The PBS was then discarded. Following the washing procedure, 100 μL of 0.5 mg/ mL of MTT reagent (Sigma-Aldrich, St. Louis, Missouri, United States) was added to each well and then incubated at 37°C for 4 hours. If formazan crystals formed, 100 μL of stopper reagent (10% sodium dodecyl sulfate in 0.1N HCl) was added to each well and incubated at 37°C for 1 hour. After the reaction was stopped, the absorbance was measured using a microplate reader (iMark; Bio-Rad, Hercules, California, United States) at 595-nm wavelength. The absorbance was recorded, and the fibroblast cell viability percentage was calculated. Data were calculated by analyzing the percentage value of fibroblast viability. 25

### Biofilm Assay

Biofilm assay was conducted with methods as reported by Widyarman and Theodorea in 2021 with modifications. 26 *P. gingivalis* ATCC and *A. actinomycetemcomitans* ATCC cultures were cultivated in a 15-mL sterile tube with brain heart infusion (Oxoid, Hampshire, United Kingdom) broth. Subsequently, 200 μL (1.5 × 10 ^8^ colony forming units/ mL) of bacterial suspension were inserted into a 96-well plate using a micropipette and incubated at 37°C for 24 hours in an anaerobic atmosphere to induce biofilm formation. Subsequently, 200 μL of different concentrations (15, 10, 7.5, 5, and 2.5%) of the cinnamon extract were added to the wells containing bacterial biofilm. In the study, 0.2% chlorhexidine was used as a positive control, and bacterial biofilm without any treatment was used as a negative control. There were two incubation times: one for 1 minute and one for 3 hours. The 1-minute incubation time was chosen because this is the gargling time of most antiseptic mouthwashes, whereas the 3-hour incubation time was intended to determine the effectiveness of the cinnamon ethanol extract in combating biofilm formation over a longer period. 18 After incubation in a 96-well plate, the plate was rinsed, dried, and stained with crystal violet solution. It was then left for 15 minutes at room temperature (±25°C). Subsequently, 200 μL of ethanol absolute were added to dissolve the crystal violet-biofilm complex residues. Biofilm turbidity was measured at 595 nm wavelength using a microplate reader (iMark; Bio-Rad, Hercules, California, United States).

### Statistical Analysis

The normality of the data was determined by the Shapiro–Wilk test. If the data were normally distributed ( *p* >0.05), a one-way analysis of variance statistical test was conducted, with a significance level of *p* <0.05. This was followed by Tukey's post hoc least significant difference test, with a significance level of *p* <0.05. The data were analyzed using Statistical Package for the Social Sciences (SPSS) software version 24 (IBM, Armonk, New York, United States).

## Results

### Phytochemical Tests Result

The results showed that the ethanol extract of cinnamon contains active compounds of flavonoids, alkaloids, saponins, tannins, quinones, and terpenoids ( Table 1 ).

**Table 1:** Phytochemical test results of the cinnamon ethanol extract (qualitative)

No	Testing type	Testing result
1	Flavonoid	+
2	Alkaloid	+
3	Saponin	+
4	Tannin	+
5	Quinone	+
6	Terpenoid	+

### Cytotoxicity Test

**Fig. 1:**
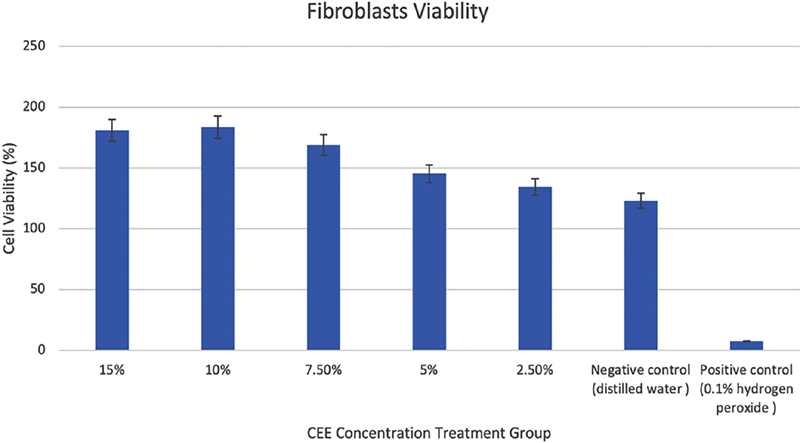
Average percentage result of fibroblasts viability using the MTT assay method after treated for 24 hours with cinnamon ethanol extract (CEE) with different concentrations (15%, 10%, 7.5%, 5%, 2.5%). 0.1% hydrogen peroxide (H _2_ O _2_ ) was used as a positive control, and sterile distilled water as a negative control.

The highest percentage of fibroblast viability was observed is the cinnamon ethanol extract treatment with a concentration of 10% (183.66 ± 18.52) and the lowest fibroblast viability was observed in the cinnamon ethanol extract with a concentration of 2.5% (134.42 ± 14.23). The process and quantity of cell death depend on material content or cell contact. 27 Different fibroblast viability in each concentration of cinnamon extract that contains ethanol can cause cells decrease which could damage the protein structure. 28 While the lowest fibroblast viability was found in the 0.1% H _2_ O _2_ as a positive control ( Fig. 1 ). Cytotoxicity tests showed that cinnamon ethanol extract did not have toxic effects on fibroblast cells (>90%). 29

### Biofilm Assay

**Fig. 2:**
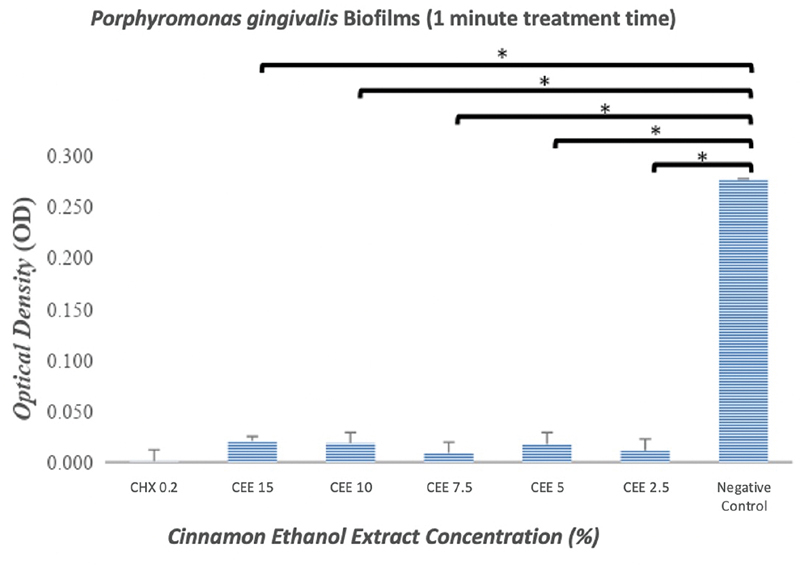
Graphs showing the effectiveness of the cinnamon ethanol extract against *Porphyromonas gingivalis* biofilm after a treatment period of 1 minute. 0.2% Chlorhexidine was used as a positive control, and bacterial biofilm without any treatment was used as a negative control. Each graph shows the average of triplicate experiments (* *p* <0.05).

**Fig. 3:**
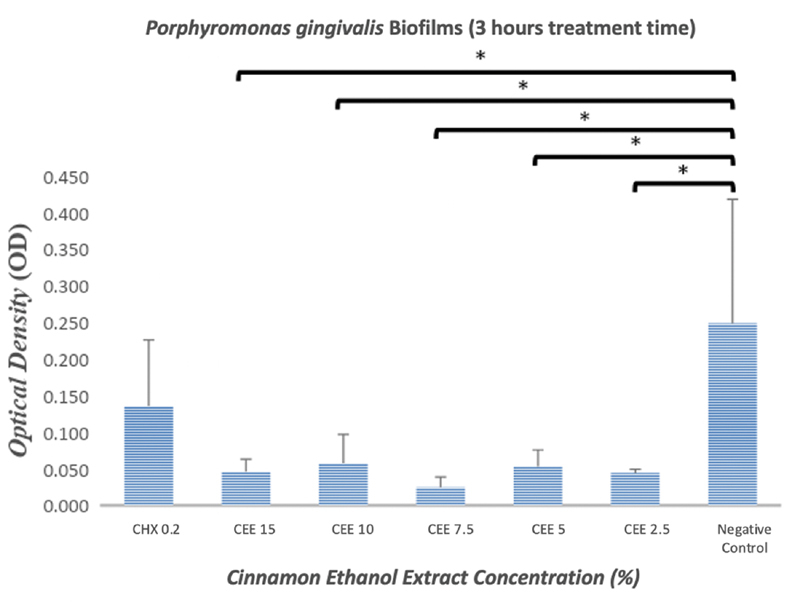
Graphs showing the effectiveness of the cinnamon ethanol extract against *Porphyromonas gingivalis* biofilm after treatment period of 3 hours. 0.2% Chlorhexidine was used as a positive control, and bacterial biofilm without any treatment was used as a negative control. Each graph shows the average of triplicate experiments (* *p* <0.05).

**Fig. 4:**
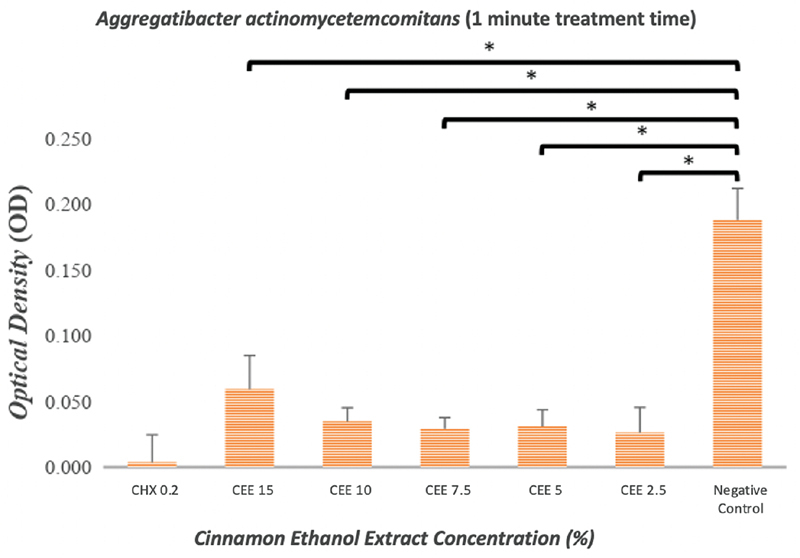
Graphs showing the effectiveness of the cinnamon ethanol extract against *Aggregatibacter actinomycetemcomitans* biofilm after a treatment period of 1 minute. 0.2% Chlorhexidine was used as a positive control, and bacterial biofilm without any treatment was used as a negative control. Each graph shows the average of triplicate experiments (* *p* <0.05).

**Fig. 5:**
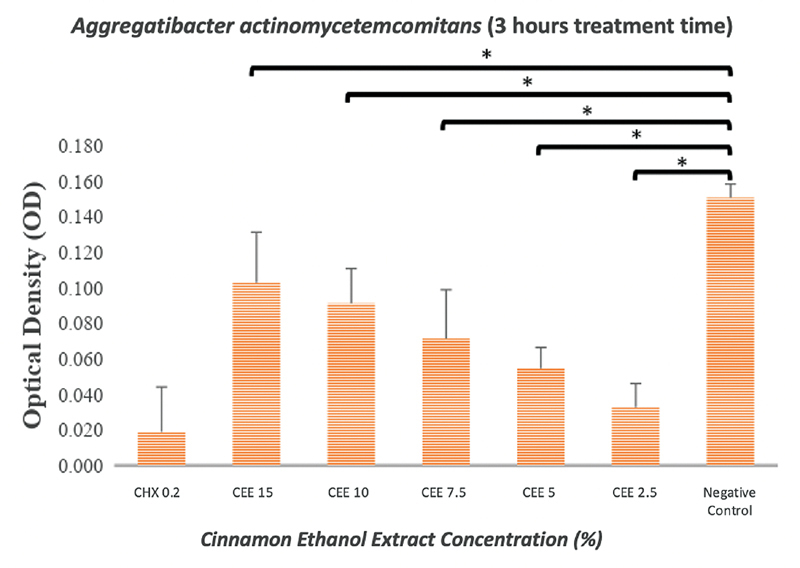
Graphs showing the effectiveness of the cinnamon ethanol extract against *Aggregatibacter actinomycetemcomitans* biofilm after treatment period of 3 hours. 0.2% Chlorhexidine was used as a positive control, and bacterial biofilm without any treatment was used as a negative control. Each graph shows the average of triplicate experiments (* *p* <0.05).

The results indicated that the ethanol extract of cinnamon exerted an antibiofilm effect against *P. gingivalis* ( Figs. 2 and 3 ) and *A. actinomycetemcomitans* ( Figs. 4 and 5 ). As shown in Figs. 2 to 5 , even the lowest concentration of cinnamon ethanol extract (2.5%) appeared to be sufficient to significantly reduce bacterial biofilms in comparison with the negative control ( *p* = 0.000).

## Discussion

Phytochemical tests were done in correlation with this study to pinpoint which phytochemical compounds are within the cinnamon ethanol extract that could exhibit antibiofilm properties. The results of the phytochemical tests confirmed that the ethanolic extract of cinnamon contained chemical compounds, including flavonoids, alkaloids, saponins, tannins, quinones, and terpenoids, as reported previously. 30 In this study ethanolic extract of cinnamon appeared to show antibiofilm activity against *P. gingivalis* and *A. actinomycetemcomitans* . This result is supported by research conducted by Raorane et al, who reported that flavonoids inhibit biofilm formation of *Acinetobacter baumannii* , as well as exhibit antivirulence activity. 31

The observed antibiofilm effects of the active compounds (flavonoids, alkaloids, and saponins) evaluated in this study are supported by research conducted by Apriliany et al In a study on *Pseudomonas aeruginosa* , they reported that flavonoids, alkaloids, and saponins in cinnamon ethanol extract exhibited an anti-quorum sensing effect, with these compounds having an adverse impact on cell–cell communication, thereby inhibiting biofilm formation. 32

In another study, essential oils containing flavonoids and tannins found in *Cinnamomum zeylanicum* and *Cinnamomum cassia* exhibited an antibiofilm effect against *Streptococcus pyogenes* , *P. aeruginosa* , and *E. coli* bacteria at a concentration of 0.2%. Electron microscopy revealed morphological changes in bacterial cells. 16 In the same study, an increase in nucleic acid and protein levels in a cell suspension pointed to cell membrane damage. The finding of flavonoids and tannins in the ethanolic extract of cinnamon in this study suggests that they play an active role in inhibiting and destroying biofilms of *P. gingivalis* and *A. actinomycetemcomitans* .

In this study, there was no significant difference in antibiofilm effect between cinnamon ethanol extract and 0.2% chlorhexidine as a positive control on the bacteria. The cinnamon ethanol extract with a concentration of 7.5% showed the most effective antibiofilm effect against *P. gingivalis* and a concentration of 2.5% was effective against *A. actinomycetemcomitans* biofilm formation. This study showed that cinnamon ethanol extract is susceptible to inhibit both the bacteria. We attributed these findings to the presence of active compounds, including flavonoids, alkaloids, saponins, tannins, quinones, and terpenoids, in the cinnamon ethanol extract. Antibiofilm activity is indicated by positive results in phytochemical tests for flavonoids, alkaloids, saponins, tannins, quinones, and terpenoids. In a previous study, Mubarak et al attributed the antibacterial activity of cinnamon to its active compounds, with the minimum inhibitory concentration around 7.5%, therefore we attempted this study to assess its antibiofilm properties using the concentration of cinnamon ethanol extract revolving around the MIC concentration. 30 The antibacterial function of flavonoids is due to their ability to form complex compounds with extracellular proteins, which disrupt the integrity of bacterial cell membranes. 33 The antibacterial activity of alkaloids is the result of their alkaline composition and effect on osmotic pressure, which can damage the bacteria cell wall and their environment. The alkaline nature of alkaloids affects the osmotic pressure between bacteria and their environment.

The antibacterial function of tannins is explained by their ability to inactivate enzymes and destroy cell membranes. The antibacterial properties of quinones are due to their ability to form complexes with amino acids, which have adverse effects on protein function. By binding to fats and carbohydrates, terpenoids have adverse effects on the permeability of the bacterial cell membrane. 34

Biofilm susceptibility to antibacterial compounds is thought to be due to several factors, including the presence of extracellular polymeric substances surrounding bacterial cells. The antimicrobial agent is absorbed into the extracellular polymeric substances and is effectively diluted in its concentration before reaching individual cells in the biofilm. 35 Based on previous study, cinnamon ethanol extracts have proved their antibacterial and antibiofilm activity to *Streptococcus pyogenes* , *Pseudomonas aeruginosa* , and *Escherichia coli* with cinnamon extract 0.2%. These findings are consistent with those of the present study where low concentrations of cinnamon ethanol extract had better antibiofilm effects than high concentrations.

In terms of the solvent and cinnamon species used, the present research is similar to a study conducted by Waty and Suryanto. In their study, they investigated the impact of ethanolic extract of cinnamon at concentrations of 6.25, 12.5, and 25% *in vitro* and reported that alkaloids, flavonoids, and saponins inhibited the growth of six species of *Streptococcus* (a gram-positive bacterium). 36 Although ethanol was used as the solvent to create the cinnamon crude extract, the ethanol will not affect the antibacterial properties of the cinnamon ethanol extract, since a properly configurated rotary evaporator will entirely evaporate the ethanol used as a solvent during maceration we hypothesized that more phytochemical compounds will be extracted using ethanol substances instead of those that are non-ethanol, which is proved to be valid by the study ( Table 1 ).

In this study, the antibiofilm effect was measured with incubation of 1 minute and 3 hours. After 3-hours treatment, a better result was recorded in cinnamon ethanol extract as an antibiofilm agent at both *P. gingivalis* and *A. actinomycetemcomitans* than 0.2% chlorhexidine ( Figs. 3 and 5 ). It is possible that in particular, 0.2% chlorhexidine has been shown to be ineffective against dental plaque in an *in vitro* model after more than 5 minutes of exposure. 38

In the present study, the results of the cytotoxicity tests of the cinnamon ethanol extract showed that the higher the concentration of the extract, the lower the mean percentage of fibroblast viability. In our study, none of the five concentrations of cinnamon ethanol extract were toxic to fibroblasts, with fibroblast viability of more than 90% observed at all concentrations as shown in Fig. 1 . In contrast, the positive control (0.1% H _2_ O _2_ ) had a very toxic effect on fibroblast viability, with viability of less than 30%. 29 In accordance with the findings of the present study, Singh et al reported that cinnamaldehyde in cinnamon ( *C. zeylanicum* ) does not have a toxic effect on fibroblast viability. 39 The findings of the present study also in accordance with Unlu et al, found that *C. zeylanicum* at concentrations of 2.5 to 20% did not show toxic effects, whereas toxic effects were observed at concentrations of 25 and 50%. 40 The finding of the present study and several other studies agree that a high concentration of cinnamon ethanol extract exhibits poor antibiofilm effectiveness. It might be caused by several phytochemicals such as glucose molecules from tannins or flavonoids contained in cinnamon ethanol extract that can increase the value of viability. However, the percentage of the viability at several concentrations could have been increased by residue on the wall of the well-plate. 41

## Conclusion

Cinnamon ethanol extract contains active compounds of flavonoids, alkaloids, saponins, tannins, quinones, and terpenoids. In this study, at high concentrations, cinnamon ethanol extract was nontoxic to fibroblasts. The cinnamon ethanol extract showed an antibiofilm effect against the periodontal pathogens *P. gingivalis* and *A. actinomycetemcomitans* . The cinnamon ethanol extract 2.5% can be recommended to be antibiofilm agent on *P. gingivalis* and *A. actinomycetemcomitans* and it was as effective as 0.2% chlorhexidine as a “gold standard” antibacterial mouthwash. Cinnamon ethanol extract might be useful as an alternative therapy for periodontal diseases. Further studies are still warranted to analyze the mechanism of action of cinnamon extract as an antibacterial agent. Moreover, *in vivo* studies are also needed to confirm the efficacy of this extract as mouthwash.
